# Regional Variation in Transplant Utilization and Agonal Times among Donation after Circulatory Death Lung Donors in the United States

**DOI:** 10.1111/ctr.70471

**Published:** 2026-01-30

**Authors:** Kentaro Noda, Jenalee N. Coster, Kunning Zhang, Alison Lafayette, Masashi Furukawa, Pablo G. Sanchez

**Affiliations:** ^1^ Section of Transplant Surgery Department of Surgery University of Chicago Biological Science Division Chicago Illinois USA; ^2^ Thoracic Surgery at St Mary's Hospital, Bon Secours Mercy Health Richmond Virginia USA; ^3^ Department of Cardiothoracic Surgery University of Pittsburgh Pittsburgh Pennsylvania USA; ^4^ Section of Thoracic Surgery Department of Surgery University of Chicago Biological Science Division Chicago Illinois USA

**Keywords:** agonal times, donation after circulatory death, donor utilization, lung transplantation

## Abstract

**Introduction:**

Utilization of donation after circulatory death (DCD) donors in lung transplant in the United States lags when compared to other countries. We sought to evaluate geographical variation in the percentage of donation after circulatory death donors, conversion to lung transplant, agonal times, and consent for donation.

**Methods:**

We queried the United Network for Organ Sharing database (2018–2023) to identify DCD donors and compared across organ procurement organizations (OPO). We illustrated the geographical variation in the location of transplant centers using DCDs, DCD donors used for solid organ transplant/ lung transplant, and median agonal time. Also, a special mapping was performed for the geographical difference of the refusal code utilization for declining the DCD lungs.

**Results:**

There were 889 DCD donors where at least one lung was used for transplant, while 13,030 (75.6%) resulted in other solid organ transplants. The utilization of DCD organs was >65% and equal across the US, while DCD transplant centers were predominantly located in 5 states. The lower utilization of DCD lungs, up to 12.7% was equally observed in each state. Over 75%, of DCDs had an agonal time ≤ 30 min in most states except South Florida, eastern Pennsylvania and Colorado/Wyoming. The map colored based on the refusal code categories also showed the geographical differences.

**Conclusion:**

As we continue to increase DCD lung utilization in the United States, better understanding of geographical barriers, adopting universal protocols in DCD organ donation and palliative care will hopefully lead to better utilization of organs from DCD donors, thereby increasing the number of transplants and decreasing organ wait times.

**Abbreviations**
BDD: brain death donors
DCD: donation after circulatory death
OPO: Organ Procurement Organization
UNOS: United Network for Organ Sharing
WIT: warm ischemic time

## 1 Introduction

Expanding equitable access to transplantation services is a current challenge and a national effort has been established focused on maximizing organ utilization with the goal of significantly increasing the total number of life‐saving transplants performed across the United States [1]. The utilization of organs from the brain death donor (BDD) population has not been improved for a decade, leaving limited untapped potential to support the nation's ambitious goals for expanding lung transplantation programs. Thus, innovative solutions are urgently needed to expand the donor pool in lung transplantation to fulfill the national commitment to dramatically increasing transplant volumes and ensuring that patients with end‐stage lung disease have meaningful access to life‐saving therapy [2].

In contrast to the resources from BDD, donation after circulatory death (DCD) donors are essential to expanding the available donor pool and have become an increasingly utilized resource in lung transplantation. Temporal trends in DCD donor utilization demonstrate a gradual adoption over time [3]. Despite this progress, significant geographic variability exists in the use of DCD lungs, and overall utilization remains relatively low in the United States, with rates around 8% as of 2021 [4]. In contrast, other countries such as the United Kingdom and Australia report substantially higher utilization rates of DCD lung donors, reflecting differing practices and policies [5]. Post‐transplant outcomes in recipients of DCD lungs are comparable to those receiving lungs from BDD, with equivalent short‐term and long‐term outcomes [4, 5, 6, 7, 8]. This suggests that the low utilization of DCD lungs in the United States is less likely to be related to concerns for post‐transplant complications but more likely reflects donor‐specific circumstances and logistical challenges.

Three major system‐level factors influencing the currently lower DCD utilization can include donor hospitals, Organ Procurement Organizations (OPOs), and transplant centers, each of which exhibit variability in practices and preferences nationwide [9, 10, 11]. The maximum time allowed from withdrawal of life support therapy to cardiac arrest varies and is defined by the policies of donor hospitals, local or regional regulations and the OPOs [10, 11]. In addition, the clinical management of patients undergoing the DCD pathway of donation remains under the potential donor healthcare team, which can lead to a high variability in practices [10, 12, 13]. This lack of standardization contributes to inconsistent practices and introduces geographical disparities in DCD utilization and dry‐run frequency [12, 14]. From the transplant perspective, a critical challenge is the occurrence of “dry‐run” [14, 15]. These arise when organs from a potential DCD donor that have been accepted and ultimately not transplanted due to factors such as a prolonged agonal time which compromises quality, the donor not expiring, or unfavorable intraoperative findings during procurement. Dry‐run cases are resource‐intensive, requiring significant allocation of medical staff, equipment, and time, all of which contribute to elevated medical costs [16, 17]. Also, previous studies have also reported that DCD donors have lower rates of consent for donation [18]. These variations may lead to biases in the number of DCD cases pursued or successfully completed across different regions in the United States.

In this study, we aimed to characterize the geographical variability in the DCD donor utilization rates and clinical practice in the donation process across the United States and to investigate the DCD lung donors who were not utilized in current transplant practice as a future possible and potential donor pool for lung transplantation.

## 2 Methods

We reviewed the United Network for Organ Sharing (UNOS) Standard Transplant Analysis and Research (STAR) deceased donor data file (data from January 2018 to March eighth, 2023 (prior to initiation of the CAS system) as well as the potential transplant recipient (PTR) match run file for the same period). All analyses were limited to donors over the age of 12 years old. We used the 12‐year‐old cutoff as some smaller adults do utilize pediatric organ donors for matching size. The study was approved by the University of Chicago (IRB25‐0862, approval date: 5/14/2025). A waiver of documentation of consent was granted for this research due to the minimal risk (Exempt Category 4 subcategory 4.i).

### 2.1 Definition of the Agonal Time of Each Controlled DCD

UNOS donor data includes several timepoints involved in the declaration of donor death and procurement, including the time of withdrawal of support, clamp, flush, and death confirmation. As previously defined [19], agonal duration was defined as the time in minutes between the start of the agonal phase (defined by UNOS as systolic blood pressure dropping below 80 mmHg, or oxygen saturation decreasing below 80%) and time of death confirmation.

### 2.2 Study Design to Investigate Geographic Distributions of the Controlled DCD Donors

For geographical comparison, the proportion of controlled DCD donor was calculated in several ways. To know the DCD organ utilization in general, the dataset was limited to controlled DCDs whose at least one major organ (hearts, lungs, livers and kidneys) was transplanted (DCD organ Tx). The organ utilization rate of DCD donors for at least one organ transplantation was calculated within the number of entire controlled DCDs. Also, the contribution of controlled DCD was determined by the number of DCD organ Tx divided by the number of the entire solid organ transplants (at least one organ was transplanted).

Next, to calculate the DCD lung utilization among the DCD donor pool, the dataset was limited to controlled DCDs whose at least one lung was transplanted (DCD lung Tx) and then the rate of DCD lungs within all controlled DCD was calculated. We then identified the number of DCD donors whose agonal time was less than or equal to 30 min. In each analysis, donors were grouped by OPO. OPOs were mapped based on the county FIPS code.

### 2.3 Descriptive Analysis

The PTR match run file was reviewed to determine reasons for the decline of lungs that were placed on the list for match to a recipient. Since each donor can have hundreds of offers prior to exhausting the list (or matching), the data were grouped by donor and then limited to the final match offer. The final match decline code was categorized as a “Warm ischemic time (WIT)”, “Logistics”, “Donor quality”, and “Others”. The codes that were used to define each category are listed in Supplemental Table S1.

### 2.4 Identifying Normothermic Regional Perfusion and Ex Vivo Lung Perfusion Cases

According to the previous reports [20], we identified cases utilizing normothermic regional perfusion (NRP) in controlled DCDs. The time frame between the pronouncement of death to cross‐clamp was calculated, and it was defined as NRP was performed if it was longer than 40 min. The cases using ex vivo lung perfusion (EVLP) were identified by using a variable for machine perfusion performed for either the left or the right lung in the STAR file.

### 2.5 Data Mapping in a US Map

A GeoJSON file for the OPTN/UNOS official GIS boundaries was obtained from a data server for the Health Resources and Services Administration (HRSA) Data Warehouse (https://gisportal.hrsa.gov/server/rest/services), then the geojson file containing all OPO/DSA polygons was converted to a sharpfile using an open‐source software (QGIS version 3.4; QGIS.org). Data mapping and geovisualization were performed using the R programming language (v. 4.5.2), leveraging packages such as sf and ggplot2. The choropleth maps were generated by assigning a fill color to the donor service area polygon of each OPO, corresponding to the data in the study design. For visual contrast and comparison between the maps, the percentage data was mapped with a fixed color scale from 0% to 100%

### 2.6 Statistical Analysis

Categorical variables were analyzed using chi‐square and Fisher exact tests; continuous variables were analyzed with Wilcoxon rank sum tests. A *p*‐value < 0.05 (two‐tailed) was used for determining statistical significance. To address the missing data in each variable, analyses were conducted using a complete case approach, including only participants with non‐missing values for the variables of interest. For variables with more than two levels or analyses with more than 2 groups, post hoc testing was performed by examining the standardized residuals for categorical variables or using Dunn's test for continuous variables. All analyses were performed using the IBM SPSS software version 31 (SPSS, Chicago IL).

## 3 Results

### 3.1 Cohort

From January 2018 to March eighth, 2023, 64,319 donors were available for transplant. Of those donors, 58,441 (90.9%) had at least one organ transplanted and 12,957 (20.1%) had at least one lung transplanted. The total number of controlled DCD donors was 17,229 (26.8% of total organ donors) and 13,919 (80.8% of DCDs) had at least one organ transplanted (DCD organ Tx); thus, the overall dry‐run rate was 19.2%. 891 DCD lung donors (6.8% of lung transplants and 5.2% of DCDs) were used for lung transplantation, and 13,039 DCD organ donors (80.9% in controlled DCDs) had no lungs used for transplantation but had at least one other organ transplanted for heart, liver and kidneys (non‐lung Tx DCDs). During the study period, we identified 205 unique zip codes for organ transplant centers using DCD organs (hearts, lungs, livers and kidneys), and their location and state level frequency are illustrated in the supplemental Figure S1. The DCD organ transplant centers are predominantly located in 5 states, California, Texas, Pennsylvania, New York and Florida, while lack of centers performed DCD organ Tx was found in Montana, Idaho and Wyoming. We also identified 54 zip codes of the transplant centers that performed lung transplantation using DCDs and their geographical distribution was mapped in Supplemental Figure S2. Similar to the geographic distribution of DCD organ Tx centers (Figure S1), California, Texas and Florida have more than a number of DCD lung transplant centers. Also, the OPTN regions #2, #9, #10 and #11 have more DCD lung transplant centers, while many of the states in the northwest and north Midwest regions (the OPTN region #6, #7 and #8) have less DCD lung transplant centers. The map in the supplemental Figure S3 illustrates the geographic distribution of DCD lung transplant cases performed in the United States and showed the number of DCD lung transplants performed in Ohio, California and North Carolina during the study period.

### 3.2 Geographical Differences in the Utilization of DCD Organs and DCD Lungs

**FIGURE 1:**
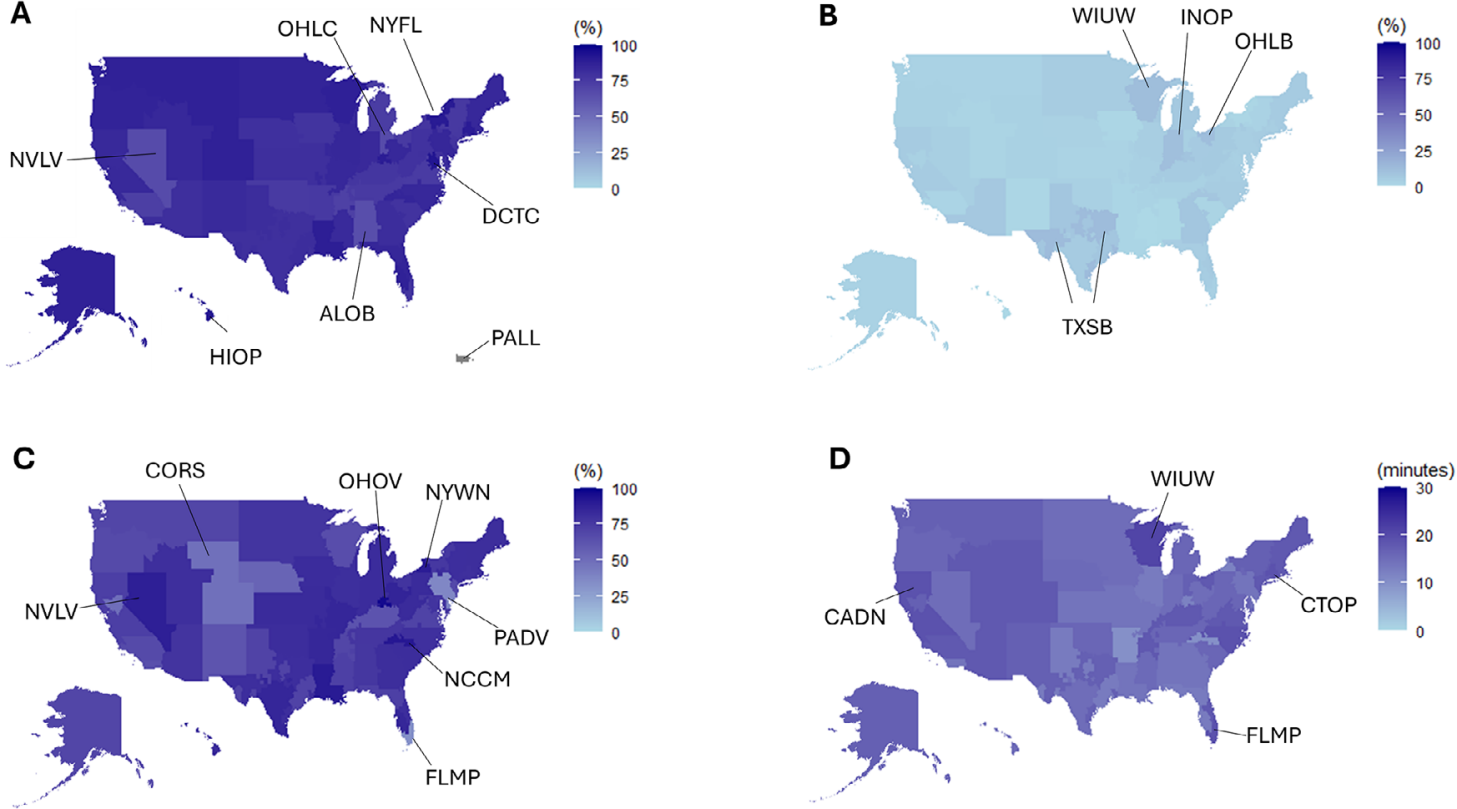
The geographic distribution of DCD utilization rate and median agonal time. The special mapping and data visualization was performed and the donor service area geometry for each Organ Procurement Organization (OPO) was colored to reflect its specific data of controlled donation after circulatory death (DCD) between 1/12018 and 3/82023 as follows: (A) The utilization rate of the DCD donors for solid organ transplantation including hearts, lung, livers and kidneys. The rate was calculated by the number of DCDs whose at least one organ was transplanted to a recipient divided by the number of all controlled DCDs available at each OPO. (B) The utilization of DCD lungs for transplantation. The rate was calculated by the number of DCDs whose at least one lung was transplanted to a recipient divided by the number of all controlled DCDs at each OPO. (C) Proportion of DCDs expired within 30 min of agonal time at each OPO. (D) Median agonal times of controlled DCDs at each OPO.

Evaluation of the proportion of DCD donors that had at least one organ transplanted showed significant geographic variation (Figure 1A). The controlled DCD utilization rates in solid organ transplantation (DCD organ Tx/controlled DCD) were at a high range. The lowest DCD utilization was 65.5% in Alabama (ALOB) followed by 65.7% in Nevada (NVLV) and 65.7% in eastern Ohio (OHLC), while the highest was 92.4% in DC (formerly covered by DCTC) followed by 91.2% in Hawaii (HIOP) and 90.2% in the Central/Upstate regions of New York state (NYFL). No controlled DCDs were identified in Puerto Rico (PALL). Regarding the contribution of controlled DCDs for overall organ transplantation (DCD organ Tx/ at least one organ transplantation, Supplemental Figure S3), DCD donors made up the highest proportion of donors who donated at least one organ in New York (NYWN 38.8%), the intermountain west region (NMOP 36.9, UTOP 37.8%, CORS 37.0%) and Oklahoma (OKOP 38.1%), whereas the proportion of DCD donors, whose at least one organ was used for transplant, were lowest in Louisiana (LAOP 12.6%), Mississippi (MSOP 11.7%) and Alabama (ALOB 11.1%).

The map in Figure 1B showed the rate of DCD lung utilization (DCD Lung Tx/controlled DCD). The range was quite lower compared to the controlled DCD utilization rates for solid organ Tx and it ranged from 0% to 12.7%. The locations of the DCD lung utilization of >10% were Texas (TXSB 12.7%), Wisconsin (WIUW 12.0), Ohio (OHLB 11.7%), and Indiana (INOP 11.1), and other OPOs had less than 10% of the lung utilization rate from their available controlled DCDs in their service area.

### 3.3 Geographical Differences in DCD Lung Allograft Donor Agonal Times

When we examined the proportion of DCD donors that had agonal times ≤ 30 min, there again was significant variability between OPOs (Figure 1C). A total of 12,861 controlled DCDs (74.7%) and 10,501 DCD organ Tx (75.4%) expired within 30 min of the agonal time. South Florida (FLMP 32.8%) had the lowest proportion of DCD donors with an agonal time ≤ 30 min followed by eastern Pennsylvania (PADV 37.4%) and Colorado/Wyoming (CORS 48.6%). In several OPOs, the agonal time was consistently ≤ 30 min. These included Nevada (NVLV 88.2%), western New York (NYWN 89.5%), and western North Carolina (NCCM 91.2%), central Ohio (formerly covered by OHOV 96.2%). A map displaying the median agonal times for each OPO is displayed in Figure 1D and the individual OPO agonal times are shown in Table 1. The median agonal time was less than 30 min across the United States, and the longest median agonal time was in Wisconsin (WIUW 21 min) followed by Connecticut (formerly covered by CTOP 19 min), south Florida (FLMP 19 min) and, north California (CADN19 min).

**TABLE 1:** Agonal times of DCD lung donors who had at least one lung transplanted.

OPO	DCD donor, n	Total donor, n	Percent DCD	Agonal time, median minutes (IQR)	Lung Tx, n	DCD, lungs used for Tx, n	Agonal time in DCD lung Tx, median minutes (IQR)
ALOB	165	1089	15.2%	14 (9, 21)	219	1	−
AROR	136	467	29.1%	9.9 (5, 15)	64	2	−
AZOB	513	1558	32.9%	18 (14, 25)	351	37	17.9 (15.0, 25)
CADN	468	1984	23.6%	19 (13, 29)	391	27	18 (13, 29)
CAGS	189	593	31.9%	17 (11.8, 31)	93	6	12.5 (8.0, −)
CAOP	564	2911	19.4%	18 (14, 24)	752	9	22 (17.5, 30.8)
CASD	206	691	29.8%	14 (9, 24)	172	15	12 (5.0, 15.0)
CORS	436	1115	39.1%	18 (13.7, 27)	188	14	20 (14, 31.5)
CTOP	66	214	30.8%	19 (15, 24)	42	3	20 (15.0167, −)
DCTC	157	683	23.0%	16 (12, 22)	181	6	18.5 (11.8, 23.5)
FLFH	300	1052	28.5%	18 (14, 25)	263	19	21 (16.0, 24.5)
FLMP	195	908	21.5%	19 (14, 26)	147	7	32 (21, −)
FLUF	210	1003	20.9%	13 (9, 18)	165	10	13 (11, 16)
FLWC	314	1473	21.3%	13 (9, 17)	262	15	15 (12.5, 19.5)
GALL	365	1818	20.1%	14 (9, 20.3)	380	30	15 (10, 21)
HIOP	34	240	14.2%	15 (10, 23)	9	0	−
IAOP	195	557	35.0%	15 (11, 22)	108	2	10.5 (2, −)
ILIP	640	2370	27.0%	13 (9, 24)	488	28	18 (9.8, 39.3)
INOP	350	1289	27.2%	15 (11, 24)	330	39	15 (9.8, 20.5)
KYDA	263	921	28.6%	18 (14, 25)	152	15	21.5 (16.8, 36.2)
LAOP	152	1125	13.5%	14 (9, 18)	235	2	11 (7, −)
MAOB	622	1928	32.3%	18 (14, 25)	309	37	18 (14, 22.5)
MDPC	139	823	16.9%	16 (11, 23)	196	6	16.5 (9, 21.0)
MIOP	741	1957	37.9%	16 (12, 23)	456	49	17 (11.3, 19.75)
MNOP	268	1005	26.7%	15 (9.7, 20)	219	15	12 (5.8, 19.3)
MOMA	402	1282	31.4%	14 (9, 21)	293	15	11 (7, 19)
MSOP	65	487	13.3%	15.5 (11, 21.7)	109	0	−
MWOB	464	1636	28.4%	14 (9, 22)	377	10	14 (9, 35.5)
NCCM	159	747	21.3%	10 (5, 15.5)	184	13	8 (5.5, 13.5)
NCNC	470	1372	34.3%	19 (14, 24)	233	25	18 (12.5, 21)
NEOR	136	394	34.5%	15.5 (11, 30.8)	62	4	14 (11, −)
NJTO	269	1123	24.0%	18 (13, 32)	155	15	18 (13.8, 33.3)
NMOP	148	365	40.5%	17 (12, 28)	25	1	−
NVLV	246	899	27.4%	15 (12, 21)	180	10	16 (10.3, 29.7)
NYAP	112	415	27.0%	17 (13, 25)	72	3	13 (9, −)
NYFL	102	303	33.7%	17 (12.5, 21)	43	1	−
NYRT	397	1839	21.6%	18 (13, 24)	297	21	18 (12.5, 21.5)
NYWN	105	246	42.7%	12 (7.5, 16)	28	3	14 (12, −)
OHLB	315	929	33.9%	18 (13, 26)	240	37	23 (15, 47)
OHLC	249	687	36.2%	17 (12, 22)	103	10	15.5 (11.2, 19.5)
OHLP	229	787	29.1%	15 (11, 20)	132	14	15.5 (12.0, 17.8)
OHOV	120	438	27.4%	12 (10, 15)	74	11	13 (7, 17)
OKOP	470	1075	43.7%	16 (10, 24)	184	17	15 (11.5, 23)
ORUO	289	875	33.0%	16 (11, 28)	138	9	13 (8, 24)
PADV	811	3307	24.5%	14 (8, 21)	510	35	27 (20.8, 64.5)
PATF	430	1473	29.2%	16 (12, 21)	250	27	17 (13.5, 24.5)
PRLL	0	521	0.0%	−	50	0	−
SCOP	320	1039	30.8%	17 (11.2, 23)	239	6	15.5 (9.8, 64.3)
TNDS	455	1916	23.7%	16 (11, 24)	373	14	15 (11.3, 22)
TNMS	82	353	23.2%	16 (11, 21.5)	55	2	17 (15, −)
TXGC	508	2085	24.4%	18 (13, 28)	538	42	17 (13, 20.3)
TXSA	265	1112	23.8%	13 (9, 19)	308	16	14 (9.3, 18.5)
TXSB	495	2132	23.2%	15 (10, 24)	608	63	15 (11, 21)
UTOP	307	750	40.9%	17 (13, 26)	104	6	18.5 (9.5, 21)
VATB	288	1078	26.7%	18 (12, 25)	219	13	18.5 (16, 23.7)
WALC	420	1530	27.5%	18 (14, 25)	281	11	16 (14, 21)
WIDN	154	537	28.7%	17 (12, 21)	121	10	21 (11.5, 29.0)
WIUW	259	813	31.9%	21 (16, 32.75)	200	31	20 (17, 25)
Total	17,229	64,319			12,957	889	

Abbreviation: OPO, Organ Procurement Organization.

DCD donor: The number of controlled DCDs available at OPO.

Total donor: The number of all organ donors available at OPO.

Percent DCD: percentage of available DCD donors in all donors at OPO.

Agonal Time: The median minutes (IQR) of the agonal time for controlled DCDs at OPO.

Lung Tx: Total number of donors whose at least one lung was used for transplantation, includes BDD and DCD donors at OPO.

DCD, lungs used for Tx: The number of controlled DCDs whose at least one lung was used for transplantation at OPO.

Agonal Time in DCD Lung Tx: The median minutes (IQR) of the agonal time for DCD Lung Tx at OPO.

### 3.4 Comparison of DCD Organs Whose Lungs Were Used and Not Used for Transplant

To know the reason for a lower utilization of DCD lungs compared to other solid organs donation from DCDs, we investigated the donor characteristics and their refusal codes for controlled DCDs whose lungs were rejected or transplanted. The comparison of the donor characteristics between the DCD lungs Tx and controlled DCD for hearts, livers and kidneys (non‐lung DCDs) are listed in Table 2. Lungs were more frequently utilized from controlled DCDs with younger age, less smoking history, and less alcohol history, compared to those in the non‐lung DCDs. Also, none‐lung DCDs found having more abnormal chest Xray, lower PO2 compared to DCD lung Tx, while DCD lung Tx had more pulmonary infection. A total 374 NRP (either abdominal or thoracoabdominal) and 361 EVLP were performed during the study period. DCD lungs include more NRP cases and more EVLP cases, compared to non‐lung DCDs. There were no statistical significances found in the percentage of the agonal time <30 min and the median agonal time between these groups.

**TABLE 2:** Comparison of donor characteristics between the controlled DCD whose lungs were transplanted or not.

			DCD, organ Tx		
Characteristic	n	Overall DCD organ Tx n = 17,229	Non‐lung DCDs n = 13,030	DCD, lungs used for Tx n = 889	*p*‐value
Donor Age (years), Median (IQR)		44 (32–54)	44 (33–54)	39 (29–50)	<0.001
Donor Sex, n (%)	13919				<0.001
Female		4638 (33.3)	4271 (32.8)	367 (41.3)	
Male		9281 (66.7)	8759 (67.2)	522 (58.7)	
Creatinine (mg/dL), Median (IQR)		0.8 (0.6–1.15)	0.8 (0.6–1.18)	0.78 (0.6–1.01)	0.078
Pulmonary Infection, n (%)	13919	8456 (60.8)	7870 (60.4)	586 (65.9)	<0.001
Cigarette History, n (%)	13919	3320 (23.9)	3261 (25)	59 (6.6)	<0.001
Heavy Alcohol History, n (%)	13919	3970 (28.5)	3758 (28.8)	212 (23.8)	0.005
Diabetes, n (%)	13807	1366 (9.9)	1283 (9.9)	83 (9.4)	0.335
Abnormal Bronchoscope, n (%)	1116	345 (30.9)	190 (39.7)	155 (24.3)	<0.001
Abnormal X‐Ray, n (%)	12708	10867 (85.5)	10264 (86.8)	603 (68.4)	<0.001
PO2 < 300, n (%)	13883	11172 (80.5)	10974 (84.5)	198 (22.3)	<0.001
Age ≥ 55 years, n (%)	13919	2741 (19.7)	2636 (20.2)	105 (11.8)	<0.001
PHS risk Donor, n (%)	13919	2882 (20.7)	2710 (20.8)	172 (19.3)	0.161
ECD, n (%)	13919	2053 (14.7)	1959 (15)	94 (10.6)	<0.001
NRP, n (%)	13919	373 (2.7)	329 (2.5)	44 (4.9)	<0.001
Machine perfusion for either lung, n (%)	3576	361 (10.1)	142 (5.2)	219 (25.2)	<0.001
Agonal time <30 min, n (%)	12332	10501 (75.4)	9835 (75.5)	666 (74.9)	0.366
Agonal time, minutes (IQR)	12332	16 (11–22)	16 (11–22)	17 (12–22.5)	0.016
Cause of Death, n (%)	13064				<0.001
Anoxia		7261 (52.2)	6915 (53.1)	346 (38.9)	
CVA		2545 (18.3)	2296 (17.6)	249 (28)	
Head Trauma		3243 (23.3)	2972 (22.8)	271 (30.5)	
CNS Tumor		15 (0.1)	15 (0.1)	0 (0)	

Abbreviations: CNS, Central Nervous System; CVA, cerebrovascular accident; ECD, Extended Criteria Donor; IQR, Interquartile Range; NRP, Normothermic Regional Perfusion; PO2, partial pressure of oxygen; WIT, warm ischemic time.

Non‐lung DCDs: DCD donors, at least one organ (heart, liver and kidney) was used for transplantation, but not lungs.

DCD, lungs used for Tx: at least one lung was used for transplantation.

The reasons for DCD lung declination are summarized in Table 3. The refusal code of DCDs for lung offers was available for 3601 cases during the study period. The most common were donor age or quality (36.2%) and the donor quality‐related concerns were also prevalent. Some were rejected by logistical concerns, including extended cold ischemic time (1.0%), organ preservation (0.6%) and long donor‐recipient distance (1.7%). Concerns about WIT and dry‐run scenarios comprised 6.1% of declines. Specifically, 219 lungs (6.1%) were declined due to uncertainty around the donor's neurological progression or failure to arrest, and 2 lungs (0.1%) due to a code: warm ischemic time too long.

**TABLE 3:** Decline reasons for DCD lung donor matches.

Refusal Reason, n (%)	N = 3601
Donor age or quality	1302 (36.2)
Donor size/weight	448 (12.5)
Unacceptable organ specific test results, specify	385 (10.7)
DCD donor neurological function/not expected to arrest	219 (6.1)
Organ size, specify	208 (5.8)
Organ‐specific donor issue	129 (3.6)
Patient txed, tx in progress, or other offer being considered	99 (2.8)
Donor medical history, specify	81 (2.3)
Donor age	72 (2.0)
Distance to travel or ship	61 (1.7)
Patient ill, unavailable, refused, or temporarily unsuitable	42 (1.2)
Candidate transplanted or pending transplant	41 (1.2)
Multiple organ transplant or different laterality is required	41 (1.2)
Actual or projected cold ischemic time too long	33 (1.0)
Organ anatomical damage or defect	25 (0.7)
Positive serological tests	23 (0.7)
Organ Preservation	19 (0.6)
Candidate temporarily medically unsuitable	16 (0.5)
Unacceptable Antigens	15 (0.5)
Positive virtual crossmatch/unacceptable antigens	14 (0.4)
PHS risk criteria or social history	14 (0.4)
Epidemic/Pandemic—Donor	11 (0.4)
Organ specific test results not available, specify	9 (0.3)
COVID‐19: OPO or transplant hospital operational issue	9 (0.3)
Candidate requires multiple organ transplant	6 (0.2)
Positive crossmatch	6 (0.2)
Number of HLA mismatches unacceptable	6 (0.2)
Donor social history	6 (0.2)
Organ preservation: Unacceptable method or findings	5 (0.2)
Positive infectious disease screening test: CMV, HBV, HCV, etc.	5 (0.2)
COVID‐19: donor‐related reason	4 (0.2)
No serum for crossmatching	3 (0.1)
Warm ischemic time too long	2 (0.1)
Candidate requires different laterality	2 (0.1)
Donor infection or positive culture	2 (0.1)
Resource time constraint (OPO, TXC, donor hospital, etc.)	2 (0.1)
Donor family time constraint	2 (0.1)
No donor cells/specimen for crossmatching, or no time for crossmatch	1 (0.1)
Operational—transplant center	1 (0.1)
Other Specify	232 (3.7)

### 3.4 Characteristics and Geographical Distribution of Refused DCD Lungs

We compared DCD donor lungs characteristics and geographical distribution among the refusal code categories for “Donor quality”, “WIT”, “Logistics” and “Others”. DCD lungs declined due to logistics appeared to be younger and have less smoking history among the groups. No differences were observed in pulmonary infection, heavy alcohol use, and diabetes among the group and even compared to those in the DCD lung Tx. (Supplemental Table S2) Overall declined donors have abnormal chest Xray more than 80%, however, about 30%–40% had abnormal bronchoscopic findings and about 50% have pO2> 300. 75.5% of non‐lung DCDs expired within 30 min and 69.9% in donor quality reason, 70.1% in logistics reason and 72.5% in other reason. Notably, 138 (62.4%) expired within 30 min of agonal time, even though lungs were refused by the WIT concerns.

**FIGURE 2:**
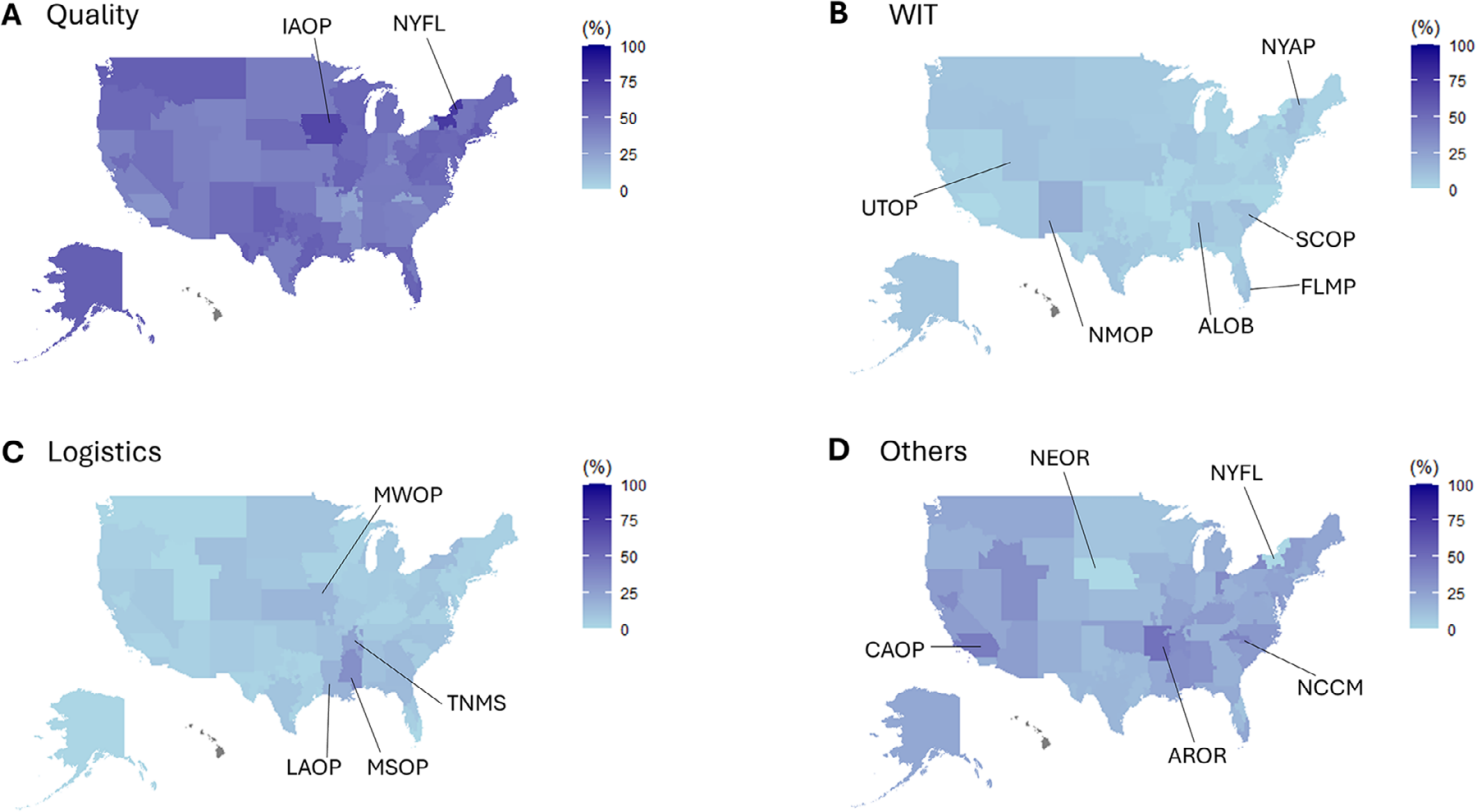
The difference in declined DCD lungs location across the United States based on the refusal code categories. The map visualizes the geographical difference of the refusal code utilization for declining DCD lungs. The utilization rate of each refusal codes category, (A) Donor quality, (B) Warm ischemic time (WIT) (C) Logistics and (D) Others, was mapped for each OPO DSA and filled with a fixed color scale.

Based on this finding, we investigate the geographic distribution of these non‐lung DCDs. The images of the spatial mapping for these refusal categories, donor quality WIT, logistics, and others, are shown in Figure 2. The DCD lung offers were declined with the donor quality related codes at 75.0% in New York State (NYFL) and 68.8% in Iowa (IAOP). (Figure 2A) The code regarding WIT was frequently (>10%) used for controlled DCDs located in New Mexico (NMOP 18.8%), the middle region of New York state (NYAP 11.5%), South Carolina (SCOP 11.1%), Alabama (ALOB 10.5%), Utah (UTOP 10.3%) and South Florida (FLMP 10.2%). (Figure 2B) Controlled DCDs in Mississippi (MSOP 33.3%), Mississippi (TNMS 23.5) Louisiana (LAOP 16.7%) and Kansas/eastern Missouri (MWOP 15.2%) were declined by logistics reasons. Other reasons were often used for DCDs in Arkansas (AROR 47.4%), the LA region of California (CAOP 42.1%), and North Carolina (NCCM 38.0), but not used for DCDs in Nebraska (NEOR) and New York State (NYFL).

## 4 Discussion

In the United States, there is a pressing need to increase the pool of available and suitable organs for lung transplant. Utilization of DCD lung allografts provides an opportunity to do so through an underutilized cohort of donors. In the present study, we found significant geographic variation in the proportion of DCD donors, their lung allograft transplant utilization and their agonal times. Most DCD donors have a short (less than 30 min) agonal time. However, within the United States, there is significant geographic variability with some regions having a high proportion of DCD donors with short agonal times, and others that have higher proportions of DCD donors with longer agonal times. Future work on standardizing donor management practices across the country could reduce the geographic variability observed in the present study and increase the pool of lungs available for transplant. Additionally, we noted a marked disparity in DCD organ utilization, with lungs being used far less frequently than other solid organs, as other studies previously showed [3, 21]. Notably, 13,030 DCD donors were used for non‐lung transplants, suggesting that a subset of these donors may have had lungs suitable for transplantation. Furthermore, our analysis revealed that a substantial number of DCD lungs were turned down without any documented reason. Targeted efforts to identify transplantable lungs among these underutilized DCD donors could contribute to a steady expansion of the donor pool for lung transplantation.

A task force from the International Society of Heart and Lung Transplant (ISHLT) identified concerns with quality, outcomes, and logistics as the main barriers for DCD donor lung utilization [16]. Similarly, this study showed the donor quality related refusal code was frequently used and it is considered mainly due to low pO2 and abnormal x‐ray. Interestingly, the majority of donors located in Iowa and a part of New York state were refused by donor quality. Also, our study found that logistical concerns, including the risk of a “dry‐run” due to the donor not expiring, are major reasons for declining DCD lungs in current practice. When evaluating logistics and resource utilization, the reported “dry run” rate associated with DCD lung donors is 40% [6, 15, 22]. While the utilization of DCD lung allografts in the United States has increased in recent years, most centers prefer to evaluate regional offers [3, 4, 23]. This is likely an attempt to avoid unnecessary travel costs if the agonal time is too long or if the donor does not expire [14]. However, data from UNOS and the ISHLT DCD database demonstrated that the majority—over 80%—of DCD donors have withdrawal and agonal times less than or equal to 30 min, while overall DCD donors in North America have longer agonal times than in other countries [7].

In another aspect, the refusal codes of cold ischemic time and traveling long distances fall into the same concern, however, recent clinical evidence demonstrated no clinical association between acute posttransplant outcomes and prolonged cold ischemic time by using a cooling device controlling the temperature during organ preservation [24, 25]. Utilizing the currently available iceless coolers may change these refusal codes for prolonged preservation time, and currently logistics‐related refused DCD lungs could be a possible donor source. Indeed, our study found Ohio, California and North Carolina as the leading states using DCD lungs, however, it also indicates that they did not always utilize their local DCD lungs based on their local DCD lung utilization (comparing between Figure 1 and S3). This suggests these leading states of DCD lung Tx may accept the donors transported from other states. Also, DCD lungs in Tennessee, Louisiana and Mississippi were frequently refused by the logistics related codes and may be suitable for transplantation.

In the present study, we found that there is significant geographical variation in the United States in agonal times, with regions in which a high percentage of DCD donors have agonal times < 30 min. Also, out of these DCD lungs declined due to the expectation of donors not expiring, our study found 75.4% did expire and at least one organ from them had been transplanted into a recipient, suggesting they could be a possible donor pool. We believe our data should encourage centers to pursue DCD lungs in regions in which there is a lower probability of a “dry run” due to higher utilization and lower agonal times. We also hope this will encourage the transplant community and policymakers to investigate the management differences that contribute to increasing utilization of lung allografts from DCD donors. In addition, the observed geographic variation in the mean agonal time may point to regional differences in end‐of‐life care. Developing a national standard for end‐of‐life care, similar to the approaches implemented in Canada and Australia [26, 27], could help balance these disparities. Such standards would ensure high‐quality comfort care for DCD donors without accelerating death, ultimately increasing the availability of transplantable lungs.

DCD lung allografts are typically transplanted at centers with higher total lung transplant volume, and there has been an increase in the proportion of centers in recent eras that utilize DCD lung donors [23]. While this suggests that higher volume centers with more experience and resources are using DCD lungs to increase their transplant volume, it is accompanied by recipients of DCD lungs having a shorter waitlist time [23]. In addition, studies analyzing differences in recipients who received DCD versus BDD lungs have noted that DCD recipients have a greater number of high‐risk factors, such as being hospitalized at time of transplant, and being bridged on extracorporeal life support [4, 6]. With survival outcomes being equivalent to BDD donors, we believe the present data should encourage most centers to consider DCD lungs for all their recipients.

There are several limitations to the current study that should be noted. First is the retrospective nature of this study, the recent practice era of the analysis of a national database and reported data. We acknowledge that our use of the UNOS database does not allow for a comprehensive assessment of all stakeholder behaviors or institutional‐level variation thus our analysis does not include the surgical factors. However, the focus of our study is to quantify the magnitude of the underutilized DCD donor pool for lungs and to identify system‐level patterns that may warrant further investigation. In addition, the UNOS data set also lacks information related to the neurological status of DCD donors which could be relevant for understanding agonal times and predicting other characteristics of likelihood of successful donation, and the refusal codes in this database are entered by different OPO coordinators, lack formal validation, potentially introducing bias into the study. Lastly, as with all studies using OPTN data, missingness remains a concern [28]. To address this, we performed complete case analyses for each variable, including only participants with non‐missing values, and reported the available sample size (n) in the tables. No imputation or other methods were applied.

In conclusion, we found that in the United States there are significant geographical differences in the proportion of DCD donors, their lung conversion to transplant and their agonal times. Our analysis may encourage national efforts for protocolized clinical management and end of life palliative care strategies for patients undergoing the DCD pathway of donation across the United States. Through understanding barriers to utilizing DCD lung donors and dispelling common misconceptions that accompany DCD donations, we hope that this underutilized resource can become a valuable asset for more transplant programs and their candidates awaiting a match.

## Funding

The authors have nothing to report.

## Disclosure

The authors have nothing to report.

## Conflicts of Interest

The authors declare no conflicts of interest.

## Supporting information




**Supporting Figure 1:** The state‐level frequency and ZIP‐code distribution of the transplant centers using organs from Donors after Circulatory Death (DCD) in the United States. State color intensity reflects the number of ZIP‐code of DCD organ transplant centers per state (light to dark blue). Red dots indicate the geographic locations of individual ZIP codes of DCD organ transplant centers.
**Supporting Figure 2:** Density and Location of Active Lung Transplant Centers using grafts from Donors after Circulatory Death (DCD) in the United States. This map displays the geographic distribution of centers where lung transplantation performed using grafts from DCD donors during the study period. The state‐level shading represents the total count of transplant centers within each state. Individual red dots pinpoint the precise geographic location of each center. This visualization identifies high‐density clusters of transplant services and highlights regions with limited access to specialized transplant facilities.
**Supporting Figure 3:** Geographic Distribution of Lung Transplantation from Donors after Circulatory Death (DCD) across the United States. The choropleth map illustrates the total number of DCD lung transplant cases performed in each state (shaded by volume). Superimposed red dots indicate the specific geographic locations of active transplant centers. Data highlights regional variations in DCD utilization and the density of transplant programs.
**Supporting Figure 4:** The contribution of DCD donors to increase the number of organ transplants. The proportion of DCD organ Tx in all donors whose at least one organ was transplanted was plotted for each OPO DSA in the US map.
**Supporting Table 1:** Refusal codes to group declined the DCD lungs.
**Supporting Table 2:** Comparison of donor characteristics among the refusal reason categories.

## Data Availability

The data that support the findings of this study are available from the corresponding author upon reasonable request.
