# Supplementary material for: Regional Variation in Transplant Utilization and Agonal Times among Donation after Circulatory Death Lung Donors in the United States
Source: Clin Transplant. 2026 Jan 30;40(2):e70471. doi: 10.1111/ctr.70471 (PMC12857602; doi:10.1111/ctr.70471)
Supplement: Supplementary file 1 — Supporting Figure 1: The state‐level frequency and ZIP‐code distribution of the transplant centers using organs from Donors after Circulatory Death (DCD) in the United States. State color intensity reflects the number of ZIP‐code of DCD organ transplant centers per state (light to dark blue). Red dots indicate the geographic locations of individual ZIP codes of DCD organ transplant centers. Supporting Figure 2: Density and Location of Active Lung Transplant Centers using grafts from Donors after Circulatory Death (DCD) in the United States. This map displays the geographic distribution of centers where lung transplantation performed using grafts from DCD donors during the study period. The state‐level shading represents the total count of transplant centers within each state. Individual red dots pinpoint the precise geographic location of each center. This visualization identifies high‐density clusters of transplant services and highlights regions with limited access to specialized transplant facilities. Supporting Figure 3: Geographic Distribution of Lung Transplantation from Donors after Circulatory Death (DCD) across the United States. The choropleth map illustrates the total number of DCD lung transplant cases performed in each state (shaded by volume). Superimposed red dots indicate the specific geographic locations of active transplant centers. Data highlights regional variations in DCD utilization and the density of transplant programs. Supporting Figure 4: The contribution of DCD donors to increase the number of organ transplants. The proportion of DCD organ Tx in all donors whose at least one organ was transplanted was plotted for each OPO DSA in the US map. Supporting Table 1: Refusal codes to group declined the DCD lungs. Supporting Table 2: Comparison of donor characteristics among the refusal reason categories. [file CTR-40-e70471-s001.docx]

**Supplemental figures and tables**

**
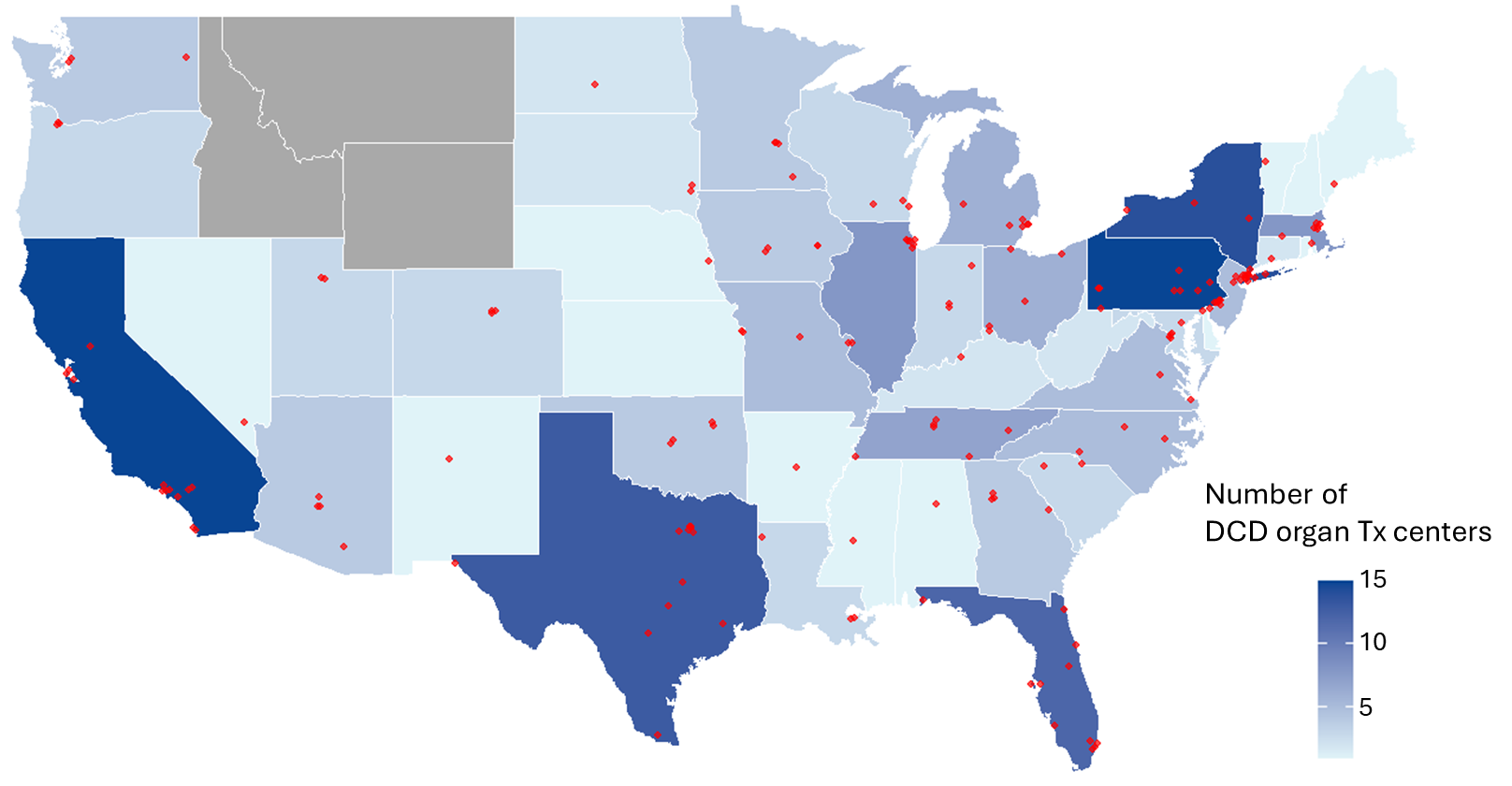
**

**Supplemental figure S1 The state-level frequency and ZIP-code distribution of the transplant centers using organs from Donors after Circulatory Death (DCD) in the United States.** State color intensity reflects the number of ZIP-code of DCD organ transplant centers per state (light to dark blue). Red dots indicate the geographic locations of individual ZIP codes of DCD organ transplant centers.


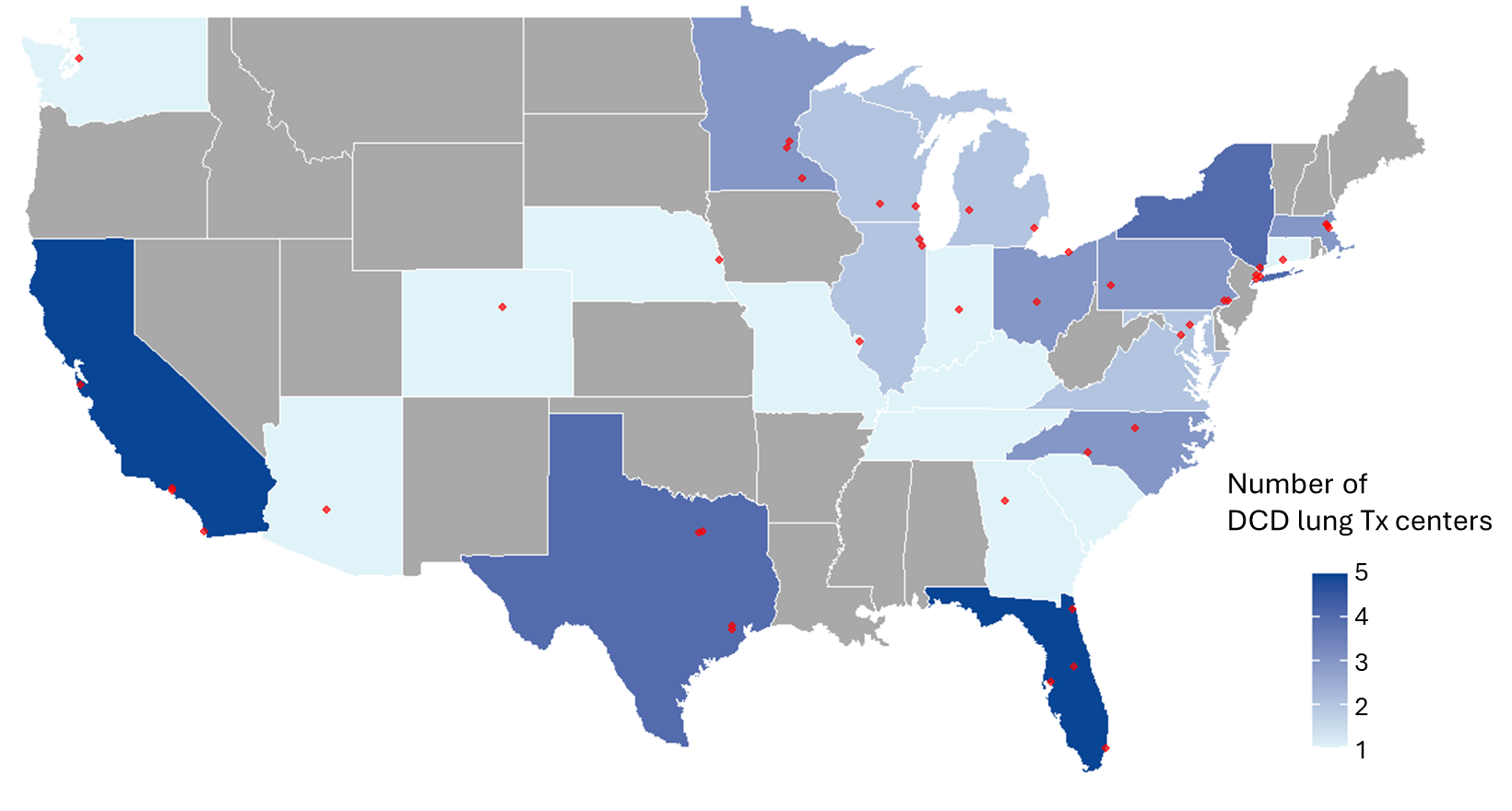


**Supplemental figure S2 Density and Location of Active Lung Transplant Centers using grafts from Donors after Circulatory Death (DCD) in the United States.** This map displays the geographic distribution of centers where lung transplantation performed using grafts from DCD donors during the study period. The state-level shading represents the total count of transplant centers within each state. Individual red dots pinpoint the precise geographic location of each center. This visualization identifies high-density clusters of transplant services and highlights regions with limited access to specialized transplant facilities.


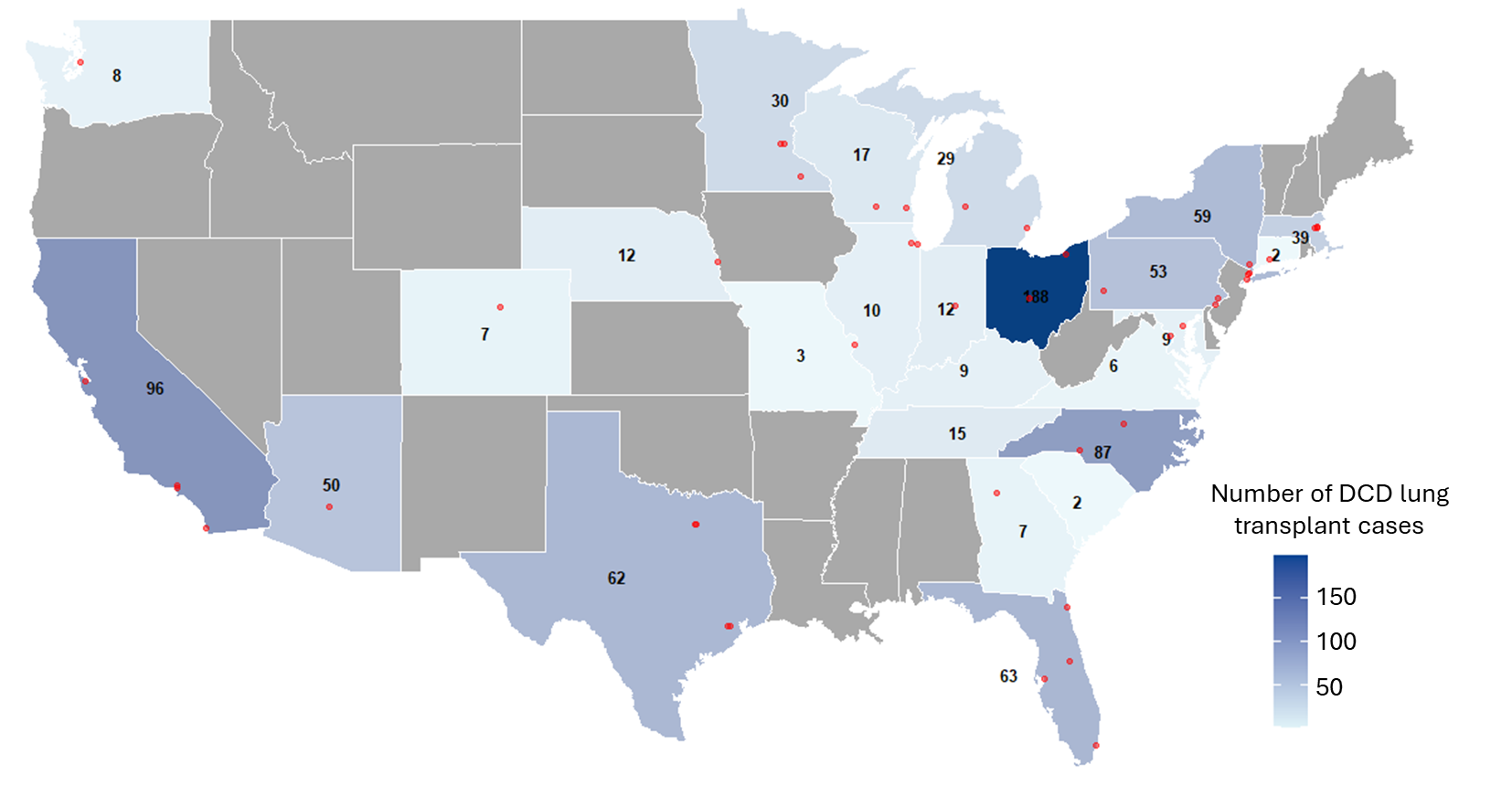


**Supplemental figure S3 Geographic Distribution of Lung Transplantation from Donors after Circulatory Death (DCD) across the United States.** The choropleth map illustrates the total number of DCD lung transplant cases performed in each state (shaded by volume). Superimposed red dots indicate the specific geographic locations of active transplant centers. Data highlights regional variations in DCD utilization and the density of transplant programs.


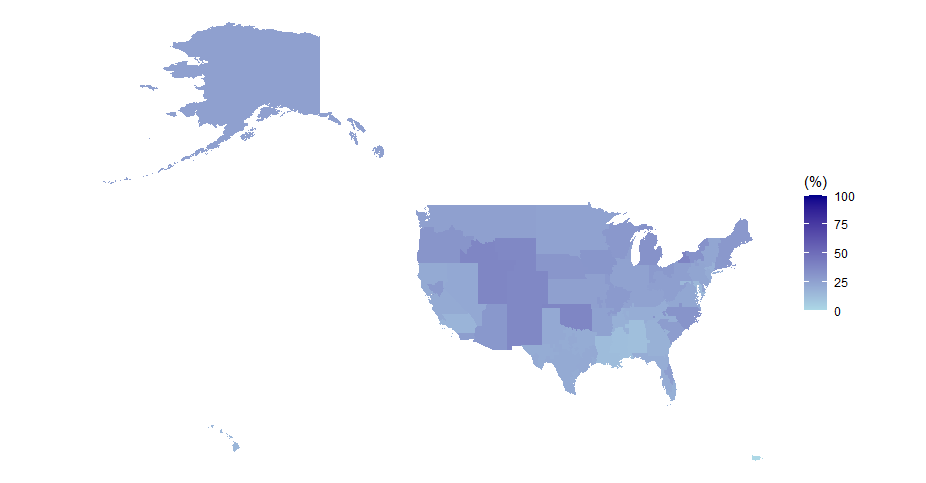

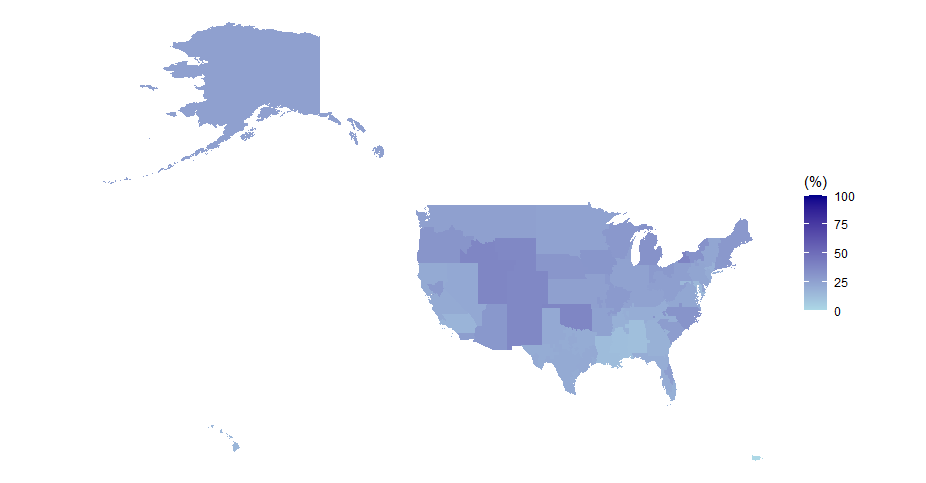

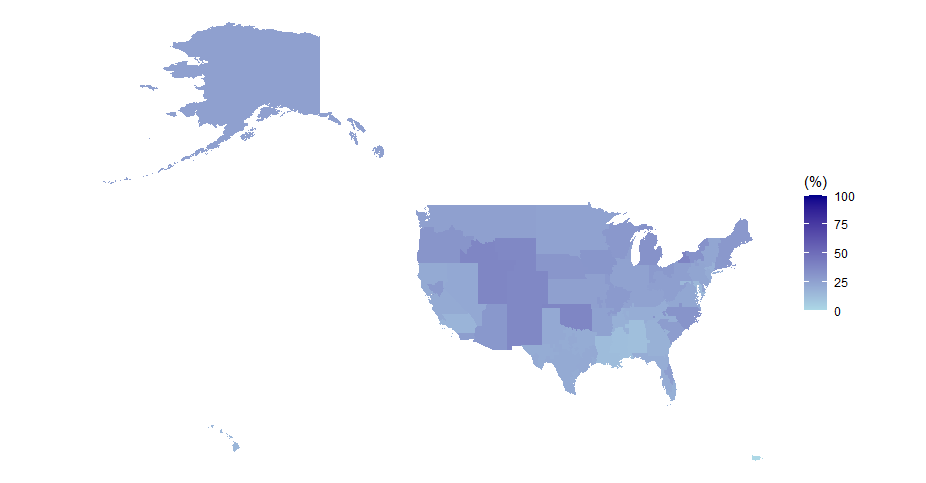


**Supplemental figure S4 The contribution of DCD donors to increase the number of organ transplants.** The proportion of DCD organ Tx in all donors whose at least one organ was transplanted was plotted for each OPO DSA in the US map.

**Supplemental Table S1. Refusal codes to group declined the DCD lungs.**

| **Group** | **Code** | **Refusal reason** |
| --- | --- | --- |
| WIT | 713 | Warm ischemic time too long |
|  | 753 | DCD donor neurological function/not expected to arrest |
| Logistics | 710 | Organ preservation: Unacceptable method or findings |
|  | 712 | Actual or projected cold ischemic time too long |
|  | 721 | Candidate transplanted or pending transplant |
|  | 724 | Candidate requires multiple organ transplant |
|  | 803 | Patient txed, tx in progress, or other offer being considered |
|  | 824 | Distance to travel or ship |
|  | 835 | Organ Preservation |
| Quality | 700 | Donor age |
|  | 701 | Organ size, specify |
|  | 740 | PHS risk criteria or social history |
|  | 830 | Donor age or quality |
|  | 831 | Donor size/weight |
|  | 837 | Organ-specific donor issue |
| Others | 711 | Organ anatomical damage or defect |
|  | 716 | Organ specific test results not available, specify |
|  | 717 | Unacceptable organ specific test results, specify |
|  | 720 | Candidate temporarily medically unsuitable |
|  | 723 | Candidate requires different laterality |
|  | 731 | No donor cells/specimen for crossmatching, or no time for crossmatch |
|  | 733 | Positive virtual crossmatch/unacceptable antigens |
|  | 741 | Positive infectious disease screening test: CMV, HBV, HCV, etc. |
|  | 742 | Donor infection or positive culture |
|  | 744 | Epidemic/Pandemic - Donor |
|  | 750 | Donor medical history, specify |
|  | 760 | Resource time constraint (OPO, TXC, donor hospital, etc.) |
|  | 761 | Donor family time constraint |
|  | 798 | Other, specify |
|  | 801 | Patient ill, unavailable, refused, or temporarily unsuitable |
|  | 802 | Multiple organ transplant or different laterality is required |
|  | 810 | Positive crossmatch |
|  | 811 | Number of HLA mismatches unacceptable |
|  | 812 | No serum for crossmatching |
|  | 813 | Unacceptable Antigens |
|  | 825 | Operational – transplant center |
|  | 833 | Donor social history |
|  | 834 | Positive serological tests |
|  | 836 | Organ anatomical damage or defect |
|  | 841 | COVID-19: donor-related reason |
|  | 842 | COVID-19: OPO or transplant hospital operational issue |
|  | 898 | Other Specify |

Table S2. Comparison of Donor Characteristics among the refusal reason categories

| **Characteristic** | **n** | **Accepted**  n= 889 | **Declined- Quality**  n= 2173 | **Declined-**  **WIT**  n= 221 | **Declined- Logistics** n= 264 | **Declined- Other**  n= 943 | **p-value**^1^ |
| --- | --- | --- | --- | --- | --- | --- | --- |
| Donor Age (years), Median (IQR) | 4490 | 39 (28 - 49) | 43 (32 - 53) | 44 (34 - 52) | 39 (29 - 50) | 41 (32 - 51) | <0.001 |
| Donor Sex, n (%) | 4490 |  |  |  |  |  | <0.001 |
| Female |  | 367 (41.3) | 700 (32.3) | 63 (28.6) | 84 (31.9) | 325 (34.5) |  |
| Male |  | 522 (58.8) | 1473 (67.8) | 158 (71.5) | 180 (68.2) | 618 (65.6) |  |
| Creatinine (mg/dL), Median (IQR) | 4490 | 0.78 (0.6 – 1.0) | 0.75 (0.6 - 1.05) | 0.72 (0.565 - 1) | 0.75 (0.6 - 1.1) | 0.78 (0.6 - 1.13) | 0.105 |
| Pulmonary Infection, n (%) | 4490 | 586 (66) | 1420 (65.4) | 154 (69.7) | 166 (62.9) | 648 (68.8) | 0.206 |
| Cigarette History, n (%) | 4398 | 59 (6.7) | 438 (20.2) | 44 (20) | 44 (16.7) | 191 (20.3) | <0.001 |
| Heavy Alcohol History, n (%) | 4385 | 212 (23.9) | 611 (28.2) | 63 (28.6) | 68 (25.8) | 262 (27.8) | 0.284 |
| Diabetes, n (%) | 4464 | 83 (9.4) | 212 (9.8) | 21 (9.5) | 26 (9.9) | 105 (11.2) | 0.745 |
| Abnormal Bronchoscope, n (%) | 1027 | 155 (24.3) | 83 (38.1) | 10 (43.5) | 10 (32.3) | 35 (29.7) | 0.001 |
| Abnormal X-Ray, n (%) | 4133 | 603 (68.5) | 1636 (83.7) | 176 (84.7) | 204 (82.6) | 703 (83.6) | <0.001 |
| PO2 < 300, n (%) | 4488 | 198 (22.3) | 1182 (54.5) | 108 (49.1) | 141 (53.5) | 536 (56.9) | <0.001 |
| Age ≥ 55 years, n (%) | 4490 | 105 (11.9) | 424 (19.6) | 43 (19.5) | 28 (10.7) | 150 (16) | <0.001 |
| PHS risk Donor, n (%) | 4490 | 172 (19.4) | 491 (22.6) | 32 (14.5) | 67 (25.4) | 210 (22.3) | 0.011 |
| ECD, n (%) | 4490 | 94 (10.6) | 352 (16.2) | 35 (15.9) | 25 (9.5) | 129 (13.7) | <0.001 |
| NRP, n (%) | 4490 | 44 (4.9) | 72 (3.3) | 16 (7.2) | 8 (3.0) | 42 (4.5) | 0.020 |
| Machine perfusion for either lung, n (%) | 1841 | 338 (18.4) | 66 (11.4) | 10 (18.2) | 13 (18.6) | 30 (11.2) | <0.001 |
| Agonal time <30min, n (%) | 4490 | 666 (74.9) | 1520 (69.9) | 138 (62.4) | 185 (70.1) | 684 (72.5) | 0.002 |
| Agonal time, minutes (IQR) |  | 17 (12 – 22.5) | 17 (12 – 26) | 18.0 (13.5- 38.5) | 17.0 (12.0-28.0) | 17.0 (12.0-24.0) | 0.005 |
| Cause of Death, n (%) | 4387 |  |  |  |  |  | <0.001 |
| Anoxia |  | 346 (39) | 1090 (50.2) | 105 (47.6) | 126 (47.8) | 508 (53.9) |  |
| CVA |  | 249 (28.1) | 496 (22.9) | 53 (24) | 52 (19.7) | 224 (23.8) |  |
| Head Trauma |  | 271 (30.5) | 543 (25) | 54 (24.5) | 77 (29.2) | 197 (20.9) |  |
| CNS Tumor |  | 0 (0) | 4 (0.2) | 1 (0.5) | 1 (0.4) | 0 (0) |  |

^1^Kruskal-Wallis rank sum test; Pearson's Chi-squared test; Pearson's Chi-squared test with simulated p-value (based on 2000 replicates); Fisher's Exact Test for Count Data with simulated p-value (based on 2000 replicates)

CNS: Central Nervous System, CVA: cerebrovascular accident, ECD: Extended Criteria Donor, IQR: Interquartile Range, NRP: Normothermic Regional Perfusion, PO2: partial pressure of oxygen, WIT: warm ischemic time
